# Nomophobia: impact of cell phone use and time to rest among teacher students

**DOI:** 10.1016/j.heliyon.2020.e04084

**Published:** 2020-05-28

**Authors:** Antonio-José Moreno-Guerrero, Jesús López-Belmonte, José-María Romero-Rodríguez, Antonio-Manuel Rodríguez-García

**Affiliations:** a University of Granada, Spain; b AREA (HUM-672) Research Group, Spain

**Keywords:** Psychology, Nomophobia, Smartphone, Teachers, Higher education, Phobia, Addiction

## Abstract

Nomophobia is one of the modern pathologies that has been born as a consequence of the impact that portable technologies have had on society and the dependence generated among citizens, especially towards smartphones. This phobia manifests itself and is intensified by the loss of immediate access to information, to the network of contacts, as well as by the impossibility of contacting or being contacted by other people. All this ends up interfering with the development of the person's daily life (physical, physiological, psychological, social problems, among others). Although the research is in an incipient phase, the problem has not been studied with the teaching population, so we present a pioneering study with this group, the main objective being to analyze the prevalence of nomophobia in future teachers of Early Childhood and Primary Education, as well as to check the incidence of rest time in the levels of nomophobia. The study follows a descriptive, correlational, transversal and predictive design and a quantitative methodology. The standardized nomophobia questionnaire NMP-Q was used on a sample of n = 849 future teachers. The results show average levels of nomophobia in most of the variables. However, the higher levels of nervousness, fear or anxiety stand out due to the inability to communicate instantaneously. Also, a higher prevalence of the problem is observed in the sector of the sample that claims to sacrifice rest time due to the use of their mobile phone. Although these numbers are not alarming, we must take into account that in some variables the prevalence is slightly higher, making it necessary to make educational interventions in this regard and to promote education for the responsible and critical use of media and technologies.

## 1 Introduction

The present manuscript attempts to analyze the levels of nomophobia in university students who are being trained to become teachers. The study also tries to know if the levels of nomophobia influence the time to rest of the future teachers. Throughout the manuscript we will present a theoretical framework. Then, the methodological procedure applied is established. The results obtained are shown below. Finally, the discussions and the main conclusions obtained in the research are shown.

The smartphone is the most used device to access the network, either to communicate with friends or family or to inquire about any aspect (Park, 2019), due to its small dimensions that facilitate portability (Barnes et al., 2019) and its high performance (Chan-Olmsted and Xiao, 2019). However, an overexposure to these devices has been generated (Ahn and Jung, 2016), as well as an inappropriate use of them, as a result of a lack of training or knowledge about them. The use of the smartphone has led to serious problems and consequences for society.

One of its consequences is nomophobia (non-mobile-phobia), which is one of the modern pathologies that has arisen as a consequence of the impact that portable technologies have had on society and the dependency generated among citizens (Anshari et al., 2019; Kneidignger-Müller, 2019). Nomophobia is one of the modern pathologies that has been born as a consequence of the impact that portable technologies have had on society and the dependence generated among citizens, especially towards smartphones. This phobia manifests itself and is intensified by the loss of immediate access to information, to the network of contacts, as well as by the impossibility of contacting or being contacted by other people (Rodríguez-García et al., 2020; Yildirim and Correia, 2015). This concept is related to the problematic use of the Internet (Ayar et al., 2018; Caplan, 2010; Moreno-Guerrero et al., 2020; Romero and Aznar, 2019) and with the increase in time dedicated to online activity and refers to an irrational fear that people have when they separate from their mobile phone (Rodríguez-García et al., 2020; Yildirim and Correia, 2015).

Although more research is required in this regard (Kneidignger-Müller, 2019), the presence of the problem is higher among young people and adolescents (Anshari et al., 2019), but it can occur at any age (Musa et al., 2017). Similarly, it is usually more common in people with low or low self-acceptance and/or self-esteem (Ozdemir et al., 2018), as well as in those who present frustrations with real-life (Arpaci et al., 2017).

According to Bragazzi and Del Puente (2014), the behavior of the nomophobic person is characterized by an obsession with having the mobile phone with themselves, always full of battery and/or carrying a charger. It is also characterized by anxiety at the mere thought of losing coverage, battery or exhaustion of their data plan. Besides, they constantly look at the screen to check the notifications and keep up to date with what is happening on the social networks and among their contacts.

Due to the paucity of studies on nomophobia, the results do not show a clear profile today of people with this problem (Adawi et al., 2019). Currently, and what can be seen today, nomophobia is directly related to the fear of not being able to communicate, lossing of connection, fear of loneliness and lossing of comfort (Ali et al., 2017). In other cases, levels of nomophobia have a strong, positive and significant relationship with difficulties in accessing the Internet, anxiety about the social profile presented on social networks, and dependence on the use of social networks (Ayar et al., 2018). In other research, it has been determined that people with nomophobia show inappropriate behaviors to cope with stress (Bragazzi et al., 2019). It has also been determined that memory, self, and proximity seeking are more common in people with high levels of nomophobia (Han et al., 2017). In other studies, three symptomatic factors have been identified as being related to this problem: anxiety, compulsive use of smart phones and feelings of panic (Musa et al., 2017). In other cases, it has been shown that nomophobia can lead to stress due to social threat, especially in cases where there is uncertainty or lack of control (Olivencia-Carrión et al., 2018).

Nomophobia can affect various population groups, especially young people, whether they are university students (Bartwal and Nath, 2019; Farooqui et al., 2017) or at other educational stages (Rosales-Huamani et al., 2019). This occurs when people are unable to communicate and are disconnected from their virtual identity. With regard to gender, some studies indicate that women show higher levels of nomophobia. This is due to their greater dependence on their smart phones (Dasgupta et al., 2017; Tams et al., 2018). Other studies, however, found no significant differences between men and women (Adawi et al., 2018; Al-Balhan et al., 2018; Lin et al., 2018; Ramos-Soler et al., 2017).

Thus, the development of nomophobia, as in the case of addictions, goes through different stages (initiation, affirmation, need and dependency) and presents multidimensional features ranging from social, physiological and physical symptoms (Anshari et al., 2019). This strong dependency, which generates feelings of anxiety, fear and/or panic when not being able to use the smartphone (Kneidignger-Müller, 2019) is due to four main factors (Yildirim and Correia, 2015): a) not being able to communicate, which refers to the feeling of not being able to communicate instantly with other people, and not being able to use the various apps and software that allow you to communicate instantly. The elements under this theme are related to the feelings of not being able to contact and be contacted; b) to lose the possibility of immediate connection, which refers to the feelings of loss of connectivity at any time and in any place, not being able to access the various social networks; c) not being able to access the information, which refers to the inconvenience of losing access to information, as well as not being able to retrieve and locate information previously displayed on their mobile phones; and d) to give up comfort, which focuses on the student's sense of comfort when they are in control of all aspects that ensure access to their mobile phone, such as battery and connectivity, among others. So the loss of access to information, the network of contacts and the inability to contact or be contacted by other people triggers a phobia that ends up causing interference in the development of the person's daily life.

The great feeling of dependence that is generated towards this device leads people to evade the real world and go into their own (Santos et al., 2017), emotional instability (Argumosa et al., 2017), attention problems (Aguilera-Manrique et al., 2018) and poor performance in studies and at work (Dasgupta et al., 2017; Mendoza et al., 2018). Additionally, this often uncontrollable dependence can lead people to sacrifice or limit their hours of sleep, food, interpersonal relationships, loss of empathy, psychosomatic symptoms such as discomfort in the bones, joints, eyes or ears, and psychological symptoms such as sadness and depression (Adawi et al., 2019; Panova et al., 2019).

In short, we are faced with a problem of a social nature that has negative consequences on people's personal and professional development. Education, as an impetus for social and economic progress, is one of the ideal means to train new generations. This requires not only training new generations with a high level of digital competence to interact effectively with the media, but the teacher themselves must be an example to follow and a reference for his students. In this way, a pioneering study is presented with the teaching community, since the literature analyzed has mainly studied health professionals (Aguilera-Manrique et al., 2018; Ahmed et al., 2019; Ayar et al., 2018) and other groups (Bragazzi et al., 2019; Gentina et al., 2018).

The teacher is a key reference for future generations in terms of their integral development and the acquisition of skills. Due to the incidence of technology in today's society and the projection of new active methodologies that involve the use of digital resources and tools (López et al., 2019), this study has focused on the population of future teachers. All this to know the state of the question about nomophobia in future professionals in the field of education. Therefore, the general objective of this research is to analyze the prevalence of nomophobia in students who are studying to become a teacher, as well as to determine the relationship between all the variables that identify the levels of nomophobia. Similarly, given that several researchers claim that nomophobia affects people's rest, this work presents the following hypotheses:

H1 Students who claim that their rest period is affected by their mobile phone users have a higher prevalence of nomophobia.

H2 More tires students have higher levels of nomophobia.

## 2 Materials and methods

The study followed the lines of a descriptive, correlational, transversal and predictive design, located within the framework of the quantitative research methodology (Hernández et al., 2016).

### 2.1 Sample

The research was carried out in the three education campuses of the University of Granada (N = 4160), specifically in the university degrees of Early Childhood Education and Primary Education from Granada (N = 3471), Ceuta (N = 368) and Melilla (N = 321). The sample size (n = 849) was selected bearing in mind a margin of error of 3%, an estimated percentage of the sample of 50% and a confidence level of 95%.

Because the study was carried out in three different cities, a stratified random sampling with proportional allocation was also applied, selecting a total of 708 students from the Granada campus, 75 students from Ceuta and 66 students from Melilla at random, in Spain. The data collection process lasted 4 months.

The sociodemographic profile of the sample was defined by 457 women (53.8%) and 392 men (46.2%), with ages ranging from 18 years (42.6%), 19 years (33.7%), 20 years (10.6%) to more 20 years (13.1%) of the Early Childhood Education Grades (44.5%) and Primary Education (55.5%).

### 2.2 Instrument

For the collection of information, the Nomophobia Questionnaire (NMP-Q scale), prepared by Yildirim and Correia (2015), and adapted to the Spanish context by González-Cabrera et al. (2017) and Gutiérrez-Puertas et al. (2016) was used. This instrument is made up of a total of 20 items, structured in four dimensions: Dimension I. Unable to communicate (6 items); Dimension II. Losing connection (5 items); Dimension III. Not being able to access the information (4 items); Dimension IV. Give up comfort (5 items). The instrument that was used was translated into Spanish, which is the language in which it was passed on to the students.

The response mode of the different items is based on a Likert-type scale from 1 to 7, with 1 being “totally in agreement” and 7 “totally disagreeing”. Total scores are calculated by adding the values of each item, giving a range of scores between 20 and 140 points. A lower score is related to a higher degree of nomophobia.

The validity and reliability values of the instrument show acceptable results (Gutiérrez-Puertas et al., 2016). The Kaiser-Meyer-Olkin statistic yielded a result of .90, the Bartlett test a value X_2190_ = 1420.8259, *p* < .01, being considered significant. Also, in the result of the initial values, four factors showed 62.7% of variance with initial values greater than 1. These results indicated the importance of the factors for the questionnaire. In the reliability test, the total Cronbach value in α was .928, presenting good internal consistency.

### 2.3 Variables

On the one hand, the independent variables of the study (predictor) were linked to the items of the NMP-Q scale. To have a better understanding in the results, the items were coded: "I would be concerned about not being able to communicate with my family and/or friends at the moment" (NMF_1); "I would worry that my family and/or friends would not be able to contact me" (NMF_2); "I would be nervous because I could not receive text messages or calls" (NMF_3); "I would be worried about not being able to keep in touch with my family and/or friends" (NMF_4); "I would be nervous because I couldn't know if someone had tried to contact me" (NMF_5); “I would be concerned that I had stopped constantly being in contact with my family and/or friends” (NMF_6); "I would be nervous about being disconnected from my virtual identity" (NMF_7); "I would feel bad for not being able to keep up to date with what is happening in the media and social networks" (NMF_8); "I would feel uncomfortable for not being able to consult the notifications about my connections and virtual networks" (NMF_9); "I would be overwhelmed by not being able to check if I have new email messages" (NMF_10); "I would feel strange because I would not know what to do" (NMF_11); "I would feel bad if I could not access the information through my smartphone at any time" (NMF_12); "It would bother me if I could not consult information through my smartphone whenever I wanted" (NMF_13); "I would be nervous if I couldn't access the news (eg events, weather forecasting, etc.) through my smartphone" (NMF_14); "It would bother me if I couldn't use my smartphone and/or its applications whenever I wanted" (NMF_15); "I would be scared if my smartphone ran out of battery" (NMF_16); “It would give me something if I was about to run out of balance or reach my monthly spending limit” (NMF_17); "If I ran out of data signal or could not connect to a Wi-Fi network, I would be constantly checking if I have recovered the signal or manage to find a network" (NMF_18); "If I could not use my smartphone, I would be afraid of being thrown somewhere" (NMF_19); "If I couldn't consult my smartphone for a while, I would feel like doing it" (NMF_20).

On the other hand, the dependent variables (criterion) were: “the incidence of mobile phone use on rest time, centered on sleeping hours” (REST) and “daily time spent on mobile phones” (UMV).

### 2.4 Procedure

The study began by contacting teaching staff from all the University of Granada campuses (Granada, Ceuta and Melilla) where the Degrees in Early Childhood and Primary Education are taught, in Spain. Also, the corresponding secretariats were asked for data on the total number of enrolled. All the collaborators who participated in the data collection did so voluntarily. These collaborators are teachers and scholarship holders from the University of Granada. All of them received specific training on research and the procedure to be followed in the collection of information.

The process of collecting information is carried out using a single-blind, with the study participants themselves not knowing the object of the research, thus trying to reduce expectations, reactivity and social desirability.

The scale was then configured using the Google Forms tool, which facilitated the collection of data from all study participants. During its application, advice was offered in case of doubts that arose during its completion. These doubts focused on purely technical aspects. That is, difficulties in connecting to the device or problems in displaying the questionnaire on the technological device used. Besides, the administration of the questionnaire was simultaneous, during school hours, to ensure homogeneity and equality of conditions.

### 2.5 Ethical considerations

The present study was approved by Ethics Committee of the AREA research group (HUM-672) of the University of Granada. Also, the study subjects offered their consent to participate in the research.

### 2.6 Data analysis

The analysis of the data was carried out using IBM SPSS version 25, in its version 25. Initially, the assumptions of linearity, independence, normality, homoskedasticity, residue analysis and non-collinearity were verified, ensuring at all times the validity of the applied statistical model (De la Fuente et al., 2018).

All the assumptions were fulfilled, except the assumptions of homoscedasticity and normality of the general linear model in certain variables. Although in the visual check of the fit of the points to the normality axis, and the skewness and kurtosis indices reflect that most of the variables did not exceed the value | 1 | and even the dimensions that exceeded it did not suppose a serious asymmetry | 2 | nor a severe kurtosis | 3 |. Therefore, it was decided to use parametric tests, since they are robust tests even when there is no serious violation of the assumptions (Montilla and Kromrey, 2010).

For the statistical study, a descriptive analysis was performed to determine the distribution of the variables, using the mean (M), standard error of measurement (SEM), standard deviation (SD), skewness and kurtosis. Bivariate Pearson correlations were also applied to check whether the linear association between the various variables of the nomophobia questionnaire is statistically significant, as well as their strength and direction. Subsequently, the descriptive statistics of comparison of means were applied, specifically the student's T-test, analyzing whether there were statistically significant differences in the variables of the nomophobia questionnaire about the incidence of smartphone use in rest time (hours of sleep). Finally, the multiple linear regression, by the stepwise method (successive steps), reported on the dependency between the variables to try to know to what extent the variables of nomophobia can be explained by the time spent on mobile phone use on the day (Giner and Soriano, 2019).

## 3 Results

The values of both skewness and kurtosis were found in a range considered adequate, showing a normal distribution of the data, given that they are between -1.96 and +1.96, as indicated by Jöreskog (2001). The kurtosis values were all negative, while the skewness values were both positive and negative. The mean of the variables was between 3 and 5, which showed that the levels of nomophobia were located in an intermediate zone (Table 1).

**Table 1:** Descriptive statistics.

Variables	M	SEM	SD	SKEWNESS	KURTOSIS
NMF_1	3.25	.069	2.011	.440	-1.007
NMF_2	3.03	.069	2.022	.644	-.855
NMF_3	4.18	.076	2.212	-.073	-1.409
NMF_4	3.28	.070	2.043	.481	-1.027
NMF_5	3.88	.071	2.082	.069	-1.272
NMF_6	3.61	.069	2.016	2.43	-1.127
NMF_7	4.60	.074	2.152	-.357	-1.267
NMF_8	4.33	.075	2.172	-.174	-1.372
NMF_9	4.39	.073	2.120	-.209	-1.311
NMF_10	4.75	.076	2.212	-.505	-1.210
NMF_11	4.29	.075	2.175	-.167	-1.359
NMF_12	4.08	.070	2.035	.007	-1.224
NMF_13	3.69	.070	2.035	.237	-1.168
NMF_14	4.46	.071	2.062	-.260	-1.206
NMF_15	3.77	.072	2.087	.173	-1.269
NMF_16	4.80	.075	2.173	-.504	-1.187
NMF_17	4.82	.076	2.208	-.530	-1.195
NMF_18	3.86	.075	2.193	.104	-1.408
NMF_19	3.79	.076	2.224	.160	-1.418
NMF_20	4.06	.073	2.114	-.017	-1303

The correlations between the independent variables, directly related to nomophobia, showed significant correlations between all of them, with a mean association strength, with exceptionalities where the association strength was low. The strongest correlations were those established between "I would be nervous because I could not receive text messages or calls" (NMF_3) and "I would be nervous because I couldn't know if someone had tried to contact me" (NMF_5); "I would be nervous about being disconnected from my virtual identity" (NMF_7) and "I would feel bad for not being able to keep up to date with what is happening in the media and social networks" (NMF_8); "I would feel bad for not being able to keep up to date with what is happening in the media and social networks" (NMF_8) and "I would feel uncomfortable for not being able to consult the notifications about my connections and virtual networks" (NMF_9); "I would be nervous about being disconnected from my virtual identity" (NMF_7) and "I would feel uncomfortable for not being able to consult the notifications about my connections and virtual networks" (NMF_9); "I would feel bad if I could not access the information through my smartphone at any time" (NMF_12) and "It would bother me if I could not consult information through my smartphone whenever I wanted" (NMF_13); "It would bother me if I could not consult information through my smartphone whenever I wanted" (NMF_13) and "It would bother me if I couldn't use my smartphone and/or its applications whenever I wanted" (NMF_15); "I would be scared if my smartphone ran out of battery" (NMF_16) and “It would give me something if I was about to run out of balance or reach my monthly spending limit” (NMF_17) (Table 2).

**Table 2:** Variable correlation.

Item	1	2	3	4	5	6	7	8	9	10	11	12	13	14	15	16	17	18	19	20
NMF_1	-																			
NMF_2	.587∗∗	-																		
NMF_3	.329∗∗	.318∗∗	-																	
NMF_4	.540∗∗	.596∗∗	.441∗∗	-																
NMF_5	.376∗∗	.375∗∗	.652∗∗	.477∗∗	-															
NMF_6	.518∗∗	.476∗∗	.457∗∗	.574∗∗	.525∗∗	-														
NMF_7	.254∗∗	.216∗∗	.539∗∗	.258∗∗	.467∗∗	.438∗∗	-													
NMF_8	.283∗∗	.241∗∗	.461∗∗	.261∗∗	.450∗∗	.422∗∗	.634∗∗	-												
NMF_9	.251∗∗	.176∗∗	.511∗∗	.257∗∗	.486∗∗	.404∗∗	.647∗∗	.656∗∗	-											
NMF_10	.126∗∗	.098∗∗	.430∗∗	.169∗∗	.381∗∗	.288∗∗	.518∗∗	.482∗∗	.540∗∗	-										
NMF_11	.233∗∗	.124∗∗	.393∗∗	.276∗∗	.381∗∗	.339∗∗	.495∗∗	.467∗∗	.494∗∗	.413∗∗	-									
NMF_12	.331∗∗	.248∗∗	.519∗∗	.310∗∗	.454∗∗	.373∗∗	.549∗∗	.541∗∗	.615∗∗	.469∗∗	.575∗∗	-								
NMF_13	.360∗∗	.313∗∗	.439∗∗	.375∗∗	.460∗∗	.435∗∗	.412∗∗	.491∗∗	.540∗∗	.324∗∗	.462∗∗	.661∗∗	-							
NMF_14	.204∗∗	.166∗∗	.385∗∗	.199∗∗	.364∗∗	.317∗∗	.476∗∗	.491∗∗	.482∗∗	.457∗∗	.435∗∗	.531∗∗	.487∗∗	-						
NMF_15	.292∗∗	.255∗∗	.450∗∗	.320∗∗	.391∗∗	.337∗∗	.458∗∗	.479∗∗	.542∗∗	.351∗∗	.435∗∗	.588∗∗	.633∗∗	.411∗∗	-					
NMF_16	.180∗∗	.109∗∗	.398∗∗	.200∗∗	.374∗∗	.278∗∗	.526∗∗	.462∗∗	.510∗∗	.411∗∗	.426∗∗	.463∗∗	.329∗∗	.396∗∗	.417∗∗	-				
NMF_17	.162∗∗	.107∗∗	3.47∗∗	.172∗∗	.296∗∗	.268∗∗	.529∗∗	.440∗∗	.499∗∗	.489∗∗	.398∗∗	.444∗∗	.325∗∗	.431∗∗	.413∗∗	.600∗∗	-			
NMF_18	.365∗∗	.295∗∗	.435∗∗	.350∗∗	.427∗∗	.362∗∗	.479∗∗	.444∗∗	.479∗∗	.291∗∗	.411∗∗	.525∗∗	.503∗∗	.348∗∗	.571∗∗	.458∗∗	.475∗∗	-		
NMF_19	.316∗∗	.274∗∗	.304∗∗	.305∗∗	.255∗∗	.302∗∗	.300∗∗	.269∗∗	.279∗∗	.173∗∗	.322∗∗	.418∗∗	.350∗∗	.283∗∗	.335∗∗	.342∗∗	.274∗∗	.409∗∗	-	
NMF_20	.282∗∗	.234∗∗	.414∗∗	.322∗∗	.374∗∗	.309∗∗	.400∗∗	.427∗∗	.482∗∗	.309∗∗	.414∗∗	.502∗∗	.486∗∗	.357∗∗	.546∗∗	.431∗∗	.409∗∗	.563∗∗	.403∗∗	-

Note: n = 849; ∗∗ = The correlation is significant at the 0.01 level (bilateral); ∗ = The correlation is significant at the 0.05 level (bilateral).

On the other hand, the analysis of the relationship established between the independent variables with the dependent variable "sleep hours affect you", offers significant relationships in all variables except "I would be concerned about not being able to communicate with my family and/or friends at the moment" (NMF_1), "I would worry that my family and/or friends would not be able to contact me" (NMF_2), "I would be nervous because I could not receive text messages or calls" (NMF_3), "I would be nervous because I couldn't know if someone had tried to contact me" (NMF_5) and "It would bother me if I couldn't use my smartphone and/or its applications whenever I wanted" (NMF_15), where it was not shown a statistically significant relationship. In the variables where the relationship was significant, the mean of the was not always above the yes, which marked that the fact of having fewer hours of sleep or rest is not associated with the fact of being able to have nomophobia, or said of another way. The fact of having nomophobia does not mean having fewer hours of rest or sleep (Table 3).

**Table 3:** Differentiated results according to the condition at rest time.

	Rest	n	M	SD	t	p
NMF_1	Yes	452	3.19	1.988	-.935	.350
	No	397	3.32	2.036		
NMF_2	Yes	452	2.95	2.048	-1.220	.223
	No	397	3.12	1.991		
NMF_3	Yes	452	4.07	2.136	-1.482	.139
	No	397	4.30	2.292		
NMF_4	Yes	452	3.11	2.019	-2.555	.011
	No	397	3.47	2.055		
NMF_5	Yes	452	3.75	2.032	-1.805	.071
	No	397	4.01	2.131		
NMF_6	Yes	452	3.45	1.952	-2.348	.019
	No	397	3.78	2.076		
NMF_7	Yes	452	4.46	2.119	-2.019	.044
	No	397	4.76	2.180		
NMF_8	Yes	452	4.07	2.141	-3.634	.000
	No	397	4.61	2.174		
NMF_9	Yes	452	4.17	2.064	-3.254	.001
	No	397	4.64	2.157		
NMF_10	Yes	452	4.50	2.221	-3.562	.000
	No	397	5.04	2.168		
NMF_11	Yes	452	4.05	2.103	-3.505	.000
	No	397	4.57	2.224		
NMF_12	Yes	452	3.91	1.964	-2.467	.014
	No	397	4.26	2.100		
NMF_13	Yes	452	3.57	1.956	-1.872	.062
	No	397	3.83	2.115		
NMF_14	Yes	452	4.30	1.996	-2.318	.021
	No	397	4.63	2.123		
NMF_15	Yes	452	3.58	1.948	-2.844	.005
	No	397	3.99	2.217		
NMF_16	Yes	452	4.50	2.207	-4.420	.000
	No	397	5.15	2.082		
NMF_17	Yes	452	4.66	2.202	-2.248	.025
	No	397	5.00	2.204		
NMF_18	Yes	452	3.60	2.177	-3.732	.000
	No	397	4.16	2.243		
NMF_19	Yes	452	3.58	2.146	-3.038	.002
	No	397	4.04	2.288		
NMF_20	Yes	452	3.73	1.984	-4.925	.000
	No	397	4.44	2.195		

For its part, the multiple linear regression model tried to identify the effect of independent variables on the time spent on mobile phone use. It was decided to apply the model of successive steps, obtaining a total of three models, where the third showed greater explanatory capacity. According to the values of the latter model, specifically, those offered by the R^2^ statistic, 7.7% of the variance in the hours you spend using the mobile phone could be explained by the variables in model 3: that is, "I would be nervous because I couldn't know if someone had tried to contact me" (NMF_5), "I would be scared if my smartphone ran out of battery" (NMF_16), "If I could not use my smartphone, I would be afraid of being thrown somewhere" (NMF_19). However, in the R^2^ corrected according to the number of variables and the subjects involved, it showed that 7.4% of the variance can be predicted by the variables indicated above (Table 4).

**Table 4:** Step regression model.

Model	R	R^2^	R^2^C	Change Statistics				
				TEE	CR2	CF	SCF	DW
1	.231	.053	.052	1.033	.053	47.728	.000	1.828
2	.263	.069	.067	1.025	.016	14.254	.000	
3	.277	.077	.074	1.022	.008	7.264	.007	

Note: TEE: Standard error of estimation; CR2: Change in R2, CF: Change in F; SCF: Simplified CF; DW: Durbin Watson.

Finally, bearing in mind the data shown in Table 5, it was determined that the variables, "I would be nervous because I couldn't know if someone had tried to contact me" (NMF_5), "I would be scared if my smartphone ran out of battery" (NMF_16) and "If I couldn't consult my smartphone for a while, I would feel like doing it" (NMF_20), which presented values less than .05, and where the results of the t-test and its critical values contribute to test the null hypothesis, being able to assume that these variables favored the explanation of the variance of the dependent variable, being those with the highest predictive capacity. Bearing in mind the B values, a marked negative relationship was collected.

**Table 5:** Coefficients of the multiple linear regression of model 3.

	B	Typed error	Beta	t	Sig.	Tolerance	VIF
3(Constant)	2.729	0.98		27.854	.000		
NMF_20	-.073	.019	-.145	-3.834	.000	.762	1.313
NMF_16	-.055	.019	-.113	-2.978	.003	.761	1.313
NMF_5	-.051	.019	-.099	-2.695	.007	.804	1.243

Note: VIF: Variance inflaction factor.

## 4 Disscusion

Several studies have shown the prevalence of nomophobia in diverse populations, especially youth and adolescents (Anshari et al., 2019) and university students (Aguilera-Manrique et al., 2018; Ahmed et al., 2019; Darvishi et al., 2019). Until now, the prevalence of this phobia has not been studied in future teachers. Thus, based on the results found, we can affirm that we are facing a generation of future teachers who present average levels of nomophobia according to the scores obtained and the standardized questionnaire interpretation code. Although these figures are not very high (Yildirim and Correia, 2015), we must bear in mind that in some variables the prevalence has been slightly higher, making it necessary to make interventions in this regard.

The nervousness or anxiety evoked by the fear of not being able to communicate is the dimension where future teachers have concentrated the highest scores. Although this is the first study to study this population, similar results have been found in other studies with university students from other specialties, as shown by the studies by Ali et al. (2017), Arpaci et al. (2017), Dasgupta et al. (2017), Gutiérrez-Puertas et al. (2019) and Mertkan et al. (2018). In this way, the impossibility that family or friends could get in contact would be the main concern for future teachers. Similarly, they would feel uneasy if they were unable to contact immediately, as well as not being able to do so regulary. Therefore, the fact of not being able to communicate is one of the factors that most worries both the population studied and those studied in the research by Betoncu and Ozdamli (2019), Correr and Bijos (2017), Gutiérrez et al. (2016) or Ramos-Soler et al. (2017). Even though immediate, synchronous and asynchronous communication is one of the great advantages that the evolution of the society led by technological innovation has brought us, we can affirm that all this is generating addiction problems (Karsay et al., 2019; Romero and Aznar, 2019), irrational fears (Mertkan et al., 2018) and absolute dependence on these devices (King et al., 2013).

On the other hand, and although it is with a moderate prevalence, it must be taken into account that future teachers claim to feel more nervous if they cannot know if someone has contacted them or because they have stopped being in daily contact with family and friends. At the same time, not being able to use or consult information from the smartphone as soon as you want, losing the data connection or Wi-Fi, as well as the fear of being left behind if the battery runs out are variables where fear, nervousness or anxiety is they increase and, therefore, being higher the level of dependency. These results reaffirm the findings found by other researchers where the need for immediate connection and the dependency generated towards smartphones is increasing (Karsay et al., 2019; King et al., 2013), especially among the younger population (Anshari et al., 2019; Rodríguez-García et al., 2020).

In addition, a very significant linear correlation coefficient has been found between the independent variables that refer to the connection with the virtual identity, keeping up to date with what is happening on social networks and the perceived discomfort for not being able to keep up to date with the notifications that receive. In this sense, investigations such as Gezgin et al. (2018) or Mertkan et al. (2018) reaffirm these results by showing the level of dependency of the young population concerning these media. This demonstrates the need for young people to be constantly connected to their virtual identity and to what is happening on the web, whether it is news from their friends, family or people they follow on social networks.

Likewise, a multiple linear correlation has been established for the discomfort felt by future teachers when they cannot consult information through the Smartphone when they want with access to it and the impossibility of using the Smartphone when they want, such and as also demonstrated by research by Gezgin, Cakir & Yildrim (2018). Therefore, we can observe a great dependence on the part of the sample regarding the constant use of the device and the need to stay permanently connected and updated regarding their social networks (fear of missing out) and communication (Fitz et al., 2019; Gutiérrez-Puertas et al., 2019; Kneidinger-Müller, 2019), as well as the different application possibilities that the smartphone offers.

Regarding the study of the dependent variable, more than half of the sample (53.4%) assured that the use they make of their smartphone affects their hours of rest. As previous research has shown (Lee et al., 2018). So the excessive use of the smartphone can cause various problems and addictions, such as social isolation, poorer nutrition, lower academic performance or work productivity, less rest time, among others (Basu et al., 2018; Dasgupta et al., 2017; Kim et al., 2017; Rodríguez-García et al., 2020). In this sense, this study showed that the sector of the sample that claimed to sacrifice hours of rest due to the excessive use that it makes of its telephone, has greater symptoms of fear, anxiety, nervousness or uneasiness due to the impossibility of contacting or being contacted by family and/or friends. As well as, for all the variables that define the dimension regarding the loss of connection (constant connection with the virtual identity, being up-to-date on social networks, and consulting notifications instantly) and where future teachers would feel strange for not knowing what to do in case this happens.

On the other hand, and in line with the results shown by Betoncu and Ozdamli (2019), the prevalence was also higher in terms of the variables that made up not being able to access the information, especially those related to a feeling of annoyance at not being able to have immediate access, the nervousness generated by not being able to keep up to date or feel that they are missing something, as well as not being able to use the different applications when desired (Kwon et al., 2016). Finally, the fear of being offline, battery or having reached the maximum monthly consumption was also higher in this percentage of the sample. Therefore, a higher prevalence of nomophobia is showed among those students who sacrifice hours of rest due to the use they make of their mobile phones.

Lastly, the t-test performed determined the three factors that presented the highest predictive capacity for the prevalence of this phobia in the study population ("I would be nervous because I couldn't know if someone had tried to contact me"; "It would bother me if I couldn't use my smartphone and/or its applications whenever I wanted"; "I would be scared if my smartphone ran out of battery").

## 5 Conclusions and future work

In summary, this research has shown that future teachers are at an average level with respect to the presence of nomophobia. For this reason, this research has various implications for the academic and scientific community. On the one hand, given that this population will be in charge of educating future generations in the safe and critical use of information and communication technologies, they must acquire high digital competence, as well as show a responsible use of the technologies. On the other hand, since most people usually spend a lot of time using their mobile phone (Barnes et al., 2019; Kwon et al., 2016), this being more common among the young population (Aljomaa et al., 2016), since prolonged and dependent use of it can cause physical symptoms (Lee et al., 2018), depression, pathological addiction, fear, anxiety (Lee et al., 2018) low productivity or poor academic performance (Duke and Montag, 2017). For this reason, it is necessary to develop prevention activities and programs that contribute to promoting controlled, conscious and responsible use from an early age, with special emphasis on teacher training. Similarly, it would be necessary to introduce educational intervention projects during the course of university studies to mitigate these problems among future teachers so that they, in turn, are able to carry them out in the classroom when they are in charge of their students.

Finally, it should be borne in mind that we are facing a study that presents some methodological limitations. First, it analyzes the perceptions of the subjects, so the degree of subjectivity of this type of non-experimental research must be considered (Hernández et al., 2016). However, it would be convenient to continue investigating this line including different variables and covering more populations, since we are facing a problem of great social impact. So from the educational and family environment, a critical and healthy use of current technology must be promoted, thus avoiding the appearance of problems such as the one studied. As future works we intend to develop new research contemplating other variables such as the level of digital competence of teachers, psychological and socio-educational variables, as well as extending the study to other contexts in order to develop contrast research. Likewise, this work serves as a precedent to develop an educational program of good practices in the use of current technologies. In addition, another interesting aspect to analyze would be if the problem of nomophobia in teachers can affect and transfer to their students in learning spaces.

## Declarations

### Author contribution statement

A-J. Moreno-Guerrero, J. López-Belmonte, J-M. Romero-Rodríguez, A-M. Rodríguez-García: Conceived and designed the experiments; Performed the experiments; Analyzed and interpreted the data; Contributed reagents, materials, analysis tools or data; Wrote the paper.

### Funding statement

This research did not receive any specific grant from funding agencies in the public, commercial, or not-for-profit sectors.

### Competing interest statement

The authors declare no conflict of interest.

### Additional information

No additional information is available for this paper.
